# Predicting Cumulative and Maximum Brain Strain Measures From HybridIII Head Kinematics: A Combined Laboratory Study and *Post-Hoc* Regression Analysis

**DOI:** 10.1007/s10439-017-1848-y

**Published:** 2017-05-11

**Authors:** Brooklynn M. Knowles, Christopher R. Dennison

**Affiliations:** Biomedical Instrumentation Laboratory, Department of Mechanical Engineering, University of Alberta, Edmonton, AB Canada

**Keywords:** Head injury, Brain injury, Brain strain, Injury biomechanics, Concussion, Helmet assessment, Sport biomechanics

## Abstract

Due to growing concern on brain injury in sport, and the role that helmets could play in preventing brain injury caused by impact, biomechanics researchers and helmet certification organizations are discussing how helmet assessment methods might change to assess helmets based on impact parameters relevant to brain injury. To understand the relationship between kinematic measures and brain strain, we completed hundreds of impacts using a 50th percentile Hybrid III head-neck wearing an ice hockey helmet and input three-dimensional impact kinematics to a finite element brain model called the Simulated Injury Monitor (SIMon) (*n* = 267). Impacts to the helmet front, back and side included impact speeds from 1.2 to 5.8 ms^−1^. Linear regression models, compared through multiple regression techniques, calculating adjusted *R* ^2^ and the *F*-statistic, determined the most efficient set of kinematics capable of predicting SIMon-computed brain strain, including the cumulative strain damage measure (specifically CSDM-15) and maximum principal strain (MPS). Resultant change in angular velocity, Δ*ω*_R_, better predicted CSDM-15 and MPS than the current helmet certification metric, peak *g*, and was the most efficient model for predicting strain, regardless of impact location. In nearly all cases, the best two-variable model included peak resultant angular acceleration, *α*_R_, and Δ*ω*_R_.

## Introduction

Brain injuries, such as concussion, occur in hockey at rates up to 0.54 for high school,^19^ 0.41–3.1 for collegiate^8^,^15^ and 1.81 for professional,^32^ per 1000 exposures. A 2012 study considering football impacts dating back to 1961 found instances of brain injuries causing disability continually increased each year.^21^ Despite the widespread use of helmets, sport and recreation-related head injury remains the second most common cause of hospitalization for traumatic brain injury (TBI).^10^ It is understood that helmet use mitigates the risk of severe focal head injury, however the perceived increase in rates of sport-related brain injuries has led to increased research efforts examining the role of helmets in brain protection. At the same time, international organizations are discussing how helmet certification methods might change towards assessing helmet ability to protect wearers from diffuse brain injury.

Minimum helmet protective capacity is currently established through standard laboratory impact testing. Acceleration,^1^,^5^ or functionals using acceleration,^29^ establish helmet ability to attenuate impact. The text in contemporary helmet standards generally does not include a rationale on the choice of attenuation metric, however it is generally accepted that the choice of head acceleration is at least partially motivated by research on head injury biomechanics dating back to the 1950s and 1960s.^2^

A group at Wayne State University performed some of the earliest work on injury thresholds in the 1960s, developing the cerebral concussion tolerance curve (WSTC). Based on animal and human exposure data,^11^ this work identified the maximum allowable linear acceleration the head can withstand a given time duration, defining a relationship between linear head acceleration and time duration and severe head injury. Efforts to approximate the WSTC inspired severity metrics that aimed to quantify impact severity using kinematics. The severity index (SI), used for football helmet certification,^29^ places a limit on the total value of resultant linear acceleration integrated over time. The Head Injury Criterion (HIC) was later developed, which also integrates linear acceleration but instead over set time duration 15 or 36 ms. Many of the helmet certification standards in use today quantify impact attenuation through peak linear acceleration (peak *g*).^1^,^4^,^5^ Helmets certified using the above approaches are credited with protecting against severe head injury in contact sports.^6^ To build on the celebrated track record of helmets in preventing severe injury, helmet certification organizations are now considering what approaches might be used to quantify impact attenuation relative to the mechanics of impact that have been suggested as relevant in diffuse injury.

As early as the 1940s, experimental work has shown angular motion to be a critical impact component causing diffuse brain injury. In 1943, Holbourn used a gelatin mixture to represent brain tissue, where resulting strains represented the occurrence of brain injuries. After applying both translational and rotational loads, he found that the greatest strains occurred under strictly rotational motion.^14^ Later, Yarnell and Ommaya subjected rhesus monkeys to whiplash conditions, confirming the significance of angular motion on brain injuries^33^ and Gennarelli *et al*. subjected squirrel monkeys to linear and angular motions, noting a greater frequency of brain lesions under head rotation.^9^ This research established a link between head rotation and diffuse brain injury.

Today, there exist assessment functions that incorporate angular kinematics, though there is no consensus over which kinematic measure or kinematic function is best for predicting diffuse injury. The generalized acceleration model for brain injury tolerance (GAMBIT^23^) and the head impact power (HIP^22^) include both linear and angular kinematics. Brain injury criterion (BrIC^30^), rotational injury criterion (RIC^17^) and power rotational injury criterion (PRHIC^17^) include only angular kinematics. The combined probability of concussion (CP) was developed as a function of linear and rotational acceleration and proved to be a better predictor for concussion than linear acceleration alone.^25^ Specific to helmet assessment, the Hockey summation of tests for the analysis of risk (Hockey STAR) formula is calculated as a function of linear and angular acceleration.^26^

Alongside efforts to establish kinematic functions to quantify impact severity, numerical head-brain models have been developed that use measures of stress and strain in brain tissue to estimate brain injury risk and distribution. A number of finite element head models exist, all with the aim to represent human tissue response to inertial loading. Developed by Takhounts *et al*. for the automotive industry, the Simulated Injury Monitor (SIMon) approximates the average male skull, cerebrospinal fluid layers, bridging veins and brain (cerebrum, cerebellum and upper spinal cord).^31^ Global Human Body Modeling Consortium (GHBMC),^30^ Wayne State University Head Injury Model (WSUHIM),^28^ and the University College Dublin Brain Trauma Model (UCDBTM)^28^ are examples of other models currently being used. In addition to the major structures of the brain and skull represented by SIMon, these models represent facial bones, scalp and in some cases a deformable skull. Computing mechanical measures such as maximum principal stress, maximum principal strain (MPS) and maximum pressure represents tissue deformation. Through correlating brain strain to injury, strain measures including the cumulative strain damage measure (CSDM) and maximum axonal strain have been proposed to represent injury risk. Numerical models have become tools used to better understand the relationship between kinematic measures and tissue strain.

Related to the ongoing discourse on kinematic functions and head-brain models, certification organizations and researchers alike are working towards improved helmet certification methods. If kinematic functionals incorporating angular head rotation are adopted, it will be necessary to use headforms that are capable of measuring head rotation, and the impact simulation method must create realistic head rotations. While the specifics of the impact test equipment are yet to be determined, it is likely that either angular head kinematics or tissue strain measures from head models will be used in future certification methods. Consequently, it is important to document how linear and angular kinematics correlate with the emerging tissue strain measures. Such documentation could ultimately influence choices on test equipment and the use of kinematic functionals as opposed to numerical head-brain models.

The objective of this work is to document correlations between measured head kinematics and tissue strain metrics using one plausible combination of test equipment and impact simulation method. Using the HybridIII head-neck and a custom impact experiment in tandem with the SIMon head-brain finite element model, we measured linear and angular head kinematics at impact and used the measures as inputs to compute brain tissue strain. The measured kinematics were then evaluated using multiple regression to determine which kinematic measures correlated with estimated brain strain and the minimum number of kinematics needed to predict strain measures.

## Materials and Methods

**Figure 1:**
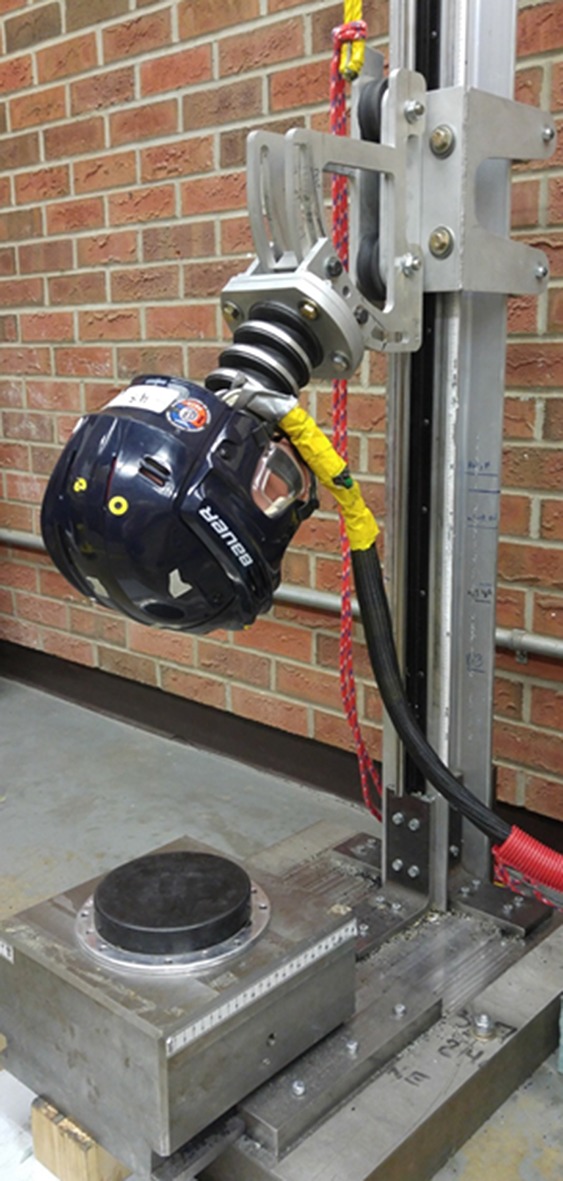
Impact tower with helmeted 50th percentile HybridIII mounted to 50th percentile neck mounted on a custom gimbal with a purpose built velocity gate.

The experimental setup included a guided rail drop tower with adjustable drop gimbal, an anthropomorphic test device (ATD) head and neck (HybridIII 50th Percentile, 10 kg total mass of gimbal and head-neck) and a modular elastomer programmer surface mounted to a stationary steel impact anvil (Fig. 1). A number of methods can be explored for simulating head impacts that allow rotation of the head and here we focused on a flexible neck approach to simulate impact. The HybridIII neck was used as it represents one model being considered for future helmet assessment methods in place of the current rigid neck set-up.^1^,^5^ It is recognized that the HybridIII neck is stiff in axial compression and another surrogate neck may ultimately be chosen, but here we represent one scenario commonly used for head impact evaluation. In future work, we will present results that consider a free drop of a head form absent a neck, similar to that being considered by European standards associations.^12^

**Figure 2:**
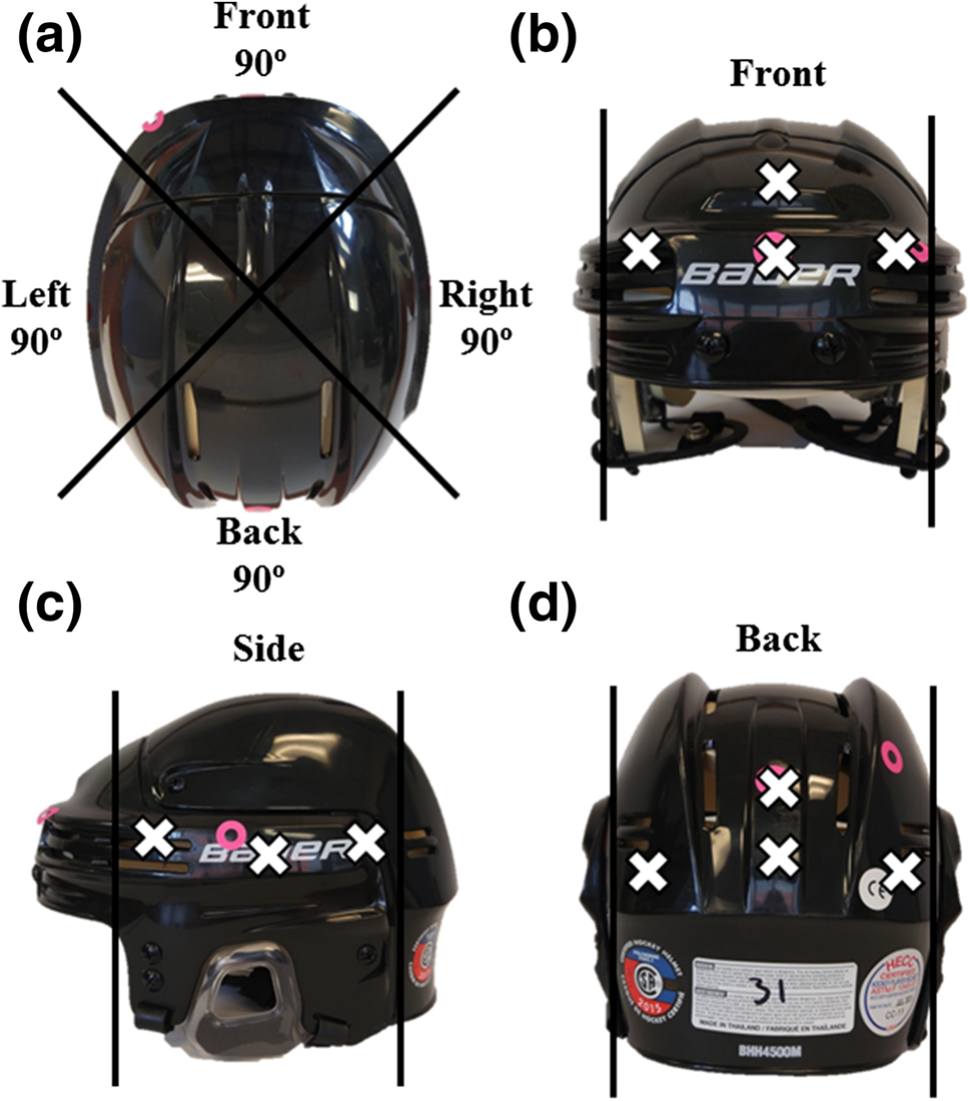
Bauer™ hockey helmet showing (a) impact regions defined by 90-degree sections as shown from the top view, and impact locations for (b) front, (c) side and (d) back impacts.

A variety of drop heights and impact locations were completed on 55 CSA certified helmets (Bauer 4500, size medium) for a total of 267 impacts. The experimental protocol was guided by common impact sites and severities experienced by collegiate ice hockey players, based on a study by Brainard *et al*. Brainard observed that the majority of impacts were to the front and back of the helmet and one percent of impacts resulted in peak linear head accelerations exceeding 80 g.^3^ In our study, 24% of impacts exceeded 80 g, which is a greater percentage than that recorded by Brainard. A greater percentage of high peak *g* events were included to capture head kinematics over a wide range of impact severities. According to Margulies *et al*., change in angular velocities exceeding 46.5 rad/s can cause diffuse axon injury (DAI).^20^ Due to the inclusion of impacts resulting in atypically high peak *g* values, a small number of impacts reached angular kinematics that can be considered within range for an individual to suffer DAI. Impact severity distribution, as quantified by peak *g*, is shown in Table 1 and impact locations referenced in this table are shown in Fig. 2. The range of impact speeds included 1.2 to 5.8 ms^−1^, which encompasses speeds specified in ice hockey helmet standards.^1^,^5^

**Table 1:** Resulting distribution of the number of impacts categorized by peak *g* range and impact location.

Location	No. of impacts			Total
	<45 g	>45 g	>80 g	
Front	43	29	33	105
Back	39	31	18	88
Side	34	28	12	74
All	116	88	63	267

Nine uniaxial accelerometers (Measurement Specialties Inc. Hampton VA, model 64C-2000-360) were mounted in the HybridIII headform, arranged in a 3-2-2-2 array. Using the conventions prescribed in Padgaonkar *et al*., we converted linear acceleration measures from the nine accelerometers to linear accelerations and angular accelerations about the head center of mass.^24^ Linear and angular velocity was computed from linear and angular acceleration, respectively using a forward integration function implemented in Matlab. Impact speed was measured by a purpose-built velocity gate setup to collect velocity data within 40 mm of impact.

Impact acceleration data was collected and saved at 100 kHz using National Instruments hardware and software (PXI 6251 and Labview v8.5, Austin TX). Analog voltages were anti-alias filtered with cut-off frequency 4 kHz using hardware prior to post-process low-pass filtering per CFC 1000.^27^

The accelerometer data collected was digitally processed using Matlab to determine the peak resultant linear acceleration and direction-specific changes in angular and linear velocity of the HybridIII headform.

HybridIII kinematics including directional linear acceleration and angular velocity were input to the Improved SIMon^31^ brain-skull FE model (solved with multi-core processor, Core™ i7-4790 CPU 8 GB RAM, Intel^®^, Santa Clara). The cumulative strain damage measure (specifically CSDM-15) and MPS are mechanical measures that here represent brain tissue deformation. CSDM-15 represents the cumulative volume fraction of the brain that reaches or exceeds a tensile strain of 15% or greater. CSDM, computed with SIMon, has corresponding risk assessment functions based on a body of injury data from animals and college football showing correlations between CSDM and probability of diffuse anatomic injury.^30^ PRHIC, based on integrated angular acceleration and angular velocity, shows strong correlation with CSDM values.^18^ MPS is capable of capturing all strain events including when strains do not exceed 15%, while the volume of brain tissue exceeding the 15% strain threshold is reported through CSDM-15. CSDM-15 and MPS are reported here as these measures were developed and correlated with injury using the SIMon model.^31^

The decision to work with SIMon, is based on the validation process done by previous researchers against cadaver and animal experiments^31^ using neutral density targets and intra-cranial pressures in order to approximate the behavior of a human brain and skull.^13^ The HybridIII head-neck setup was used during validation, making SIMon an appropriate choice for our experimental set-up.

Three-dimensional data from the 267 impacts were input to SIMon (simulation time needed to reach convergence being approximately 2–3 h per simulation). CSDM-15 and MPS were determined over 80 ms. impact duration, allowing both CSDM-15 and MPS to reach a stable maximum. Here, SIMon computed CSDM-15 and MPS are relative measures for brain strain and therefore increases in strain measures are considered to indicate greater risk of brain injury.

To determine the most efficient set of kinematics that predict SIMon computed brain strain, including CSDM-15 and MPS, linear regression models were compared through multiple regression techniques using the equations below.

$${\text{CSDM}}15 = a_{0} + \beta_{1} a_{1} + \beta_{2} a_{2} + \beta_{3} a_{3} + \cdots + \beta_{k} a_{k}$$

$${\text{MPS}} = c_{0} + \varepsilon_{1} c_{1} + \varepsilon_{2} c_{2} + \varepsilon_{3} c_{3} + \cdots + \varepsilon_{k} c_{k}.$$

Beginning with a single predictor (*k* = 1), as the linear model evolved by adding or replacing kinematic terms (a_k_, c_k_), statistical measures including weighted coefficients (*β*_*k*_ *, ε*_*k*_) and their significance (*p* < *0.05*) and adjusted *R* ^2^ were computed. For a given number of predictor terms, the magnitude of *R* ^2^ conveys which model best predicts variation in CSDM-15 and MPS. *R* ^2^ will always increase with added terms, and therefore adjusted *R* ^2^ was computed to account for the increased number of terms.^7^ To allow comparison of models with similar *R* ^2^ values, the *F*-statistic was calculated. The *F*-statistic here was used to represent how efficiently the model predicts the data set and, similar to *R* ^2^, a higher *F*-statistic is favorable.^7^

Multiple Regression analysis allowed us to compare all models and identify which kinematics were significant predictors (*p* < *0.05*), which model best fit the data (*R* ^2^) and determine if this was the most efficient model to predict the data (*F*). An ideal model would have a maximum *F*-statistic with *R* ^2^ close to 1 and all predictor variables showing significance.

**Figure 3:**
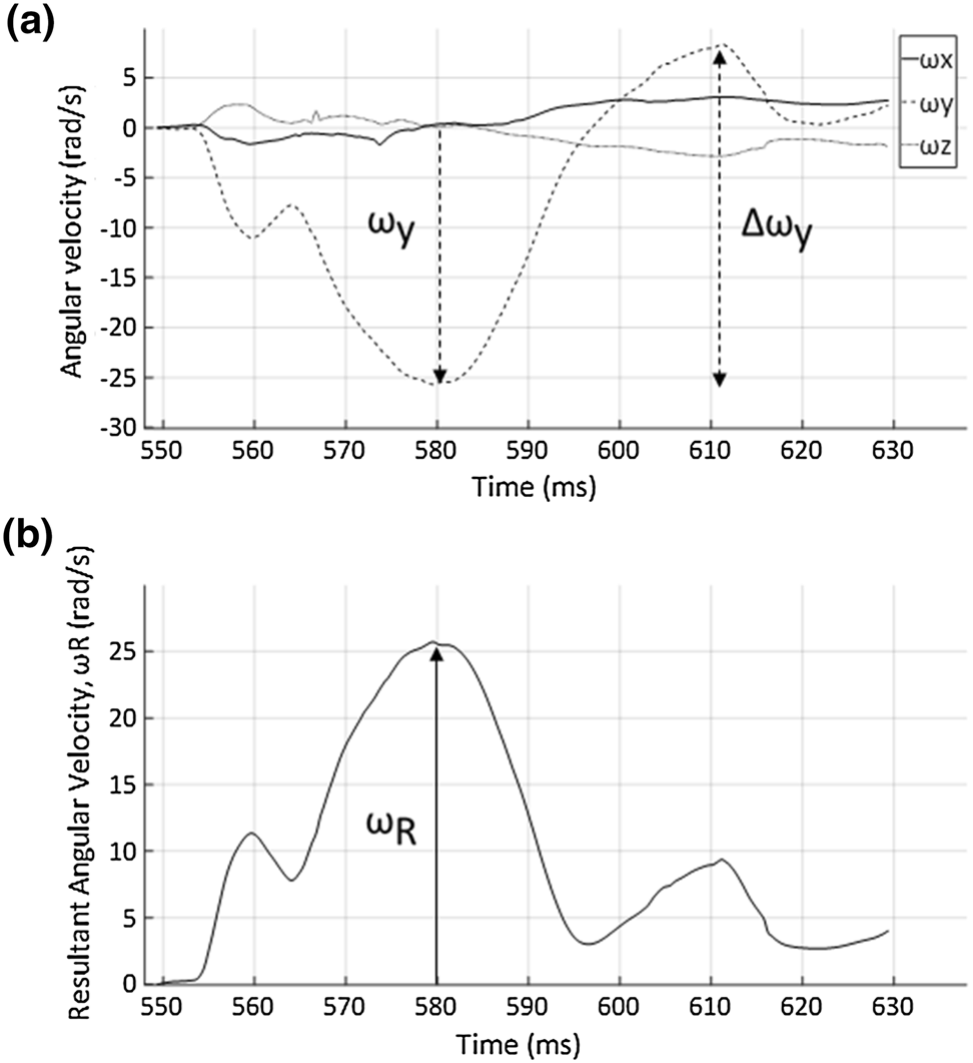
Angular velocity plotted over time: (a) Three-dimensional angular velocity demonstrating the difference between *ω*y and Δ*ω*y; Δ*ω*x and Δ*ω*z were determined in the same way and Δ*ω*_R_ was calculated as the resultant of these three values; (b) Resultant angular velocity over the impact time duration used to determine *ω*_R_.

The single kinematics that will be considered individually and in combination include: peak resultant linear acceleration (peak *g*), impact velocity (*V*_i_), resultant change in linear velocity (Δ*V*_R_), peak resultant linear velocity (*V*_R_), peak resultant angular acceleration (*α*_R_), resultant change in angular velocity (Δ*ω*_R_), directional change in angular velocity (Δ*ω*_x_, Δ*ω*_y,_ Δ*ω*_z_), peak resultant angular velocity (*ω*_R_) and directional peak angular velocity (*ω*_x_, *ω*_y_, *ω*_z_). Figure 3 contains example plots of angular velocity to demonstrate how kinematic terms were defined (e.g. resultant vs. direction-specific). Maximum kinematic values were determined irrespective of the time that the values occurred. The conventions displayed in Fig. 3 for determining change in resultant and peak angular velocity values were the same as those used for linear velocity and linear acceleration.

## Results

Results for multiple regression models predicting CSDM-15 and MPS, considering all impact locations together, are found in Tables 2 and 3, respectively. Multiple regression results specific to impact location, considering separately impacts to the front, back and side, have been summarized in Tables 4, 5, and 6. The regression models that achieved the maximum *F*-statistic, maximum adjusted *R* ^2^, and the best two-variable regression model are indicated in Tables 4, 5, and 6, respectively, presenting which variables were used to create each model based on impact location.

**Table 2:** Multiple regression models for CSDM-15 with each row containing a unique set of predictor variables to form a model with the model adjusted coefficient of determination (Adj *R* ^2^) and *F* value in the right hand columns (bold text in adjusted *R* ^2^ and *F* columns indicate maximum values).

No. of variables	Model no.	Peak *g*	*V*_i_	Δ*V*_R_	*V*_R_	*α*_R_	Δ*ω*x	Δ*ω*y	Δ*ω*z	Δ*ω*_R_	*ω*x	*ω*y	*ω*z	*ω*_R_	Adj *R* ^2^	*F*
1	1	***0.004***													0.36	152
	2		***0.096***												0.40	181
	3			***0.090***											0.44	210
	4				***0.101***										0.46	229
	5									***0.017***					0.86	**1629**
	6													***0.022***	0.82	1252
	7					***0.000***									0.11	33
2	8	−0.002		***0.120***											0.44	107
	9	−***0.003***			***0.164***										0.48	123
	10	−***0.001***								***0.018***					0.86	841
	11	***0.001***												***0.020***	0.83	658
	12			−0.001						***0.017***					0.86	812
	13			***0.025***										***0.019***	0.85	731
	14				***0.026***									***0.019***	0.84	713
	15				−0.003					***0.017***					0.86	813
	16	***0.005***				0.000									0.37	75
	17			***0.096***		0.000									0.46	106
	18				***0.110***	0.000									0.48	116
	19					***0.000***				***0.017***					0.87	851
	20					0.000								***0.022***	0.83	621
3	21	−***0.003***		***0.052***						***0.017***					0.88	638
	22	−***0.002***		***0.071***										***0.019***	0.86	544
	23	−***0.003***			***0.054***					***0.017***					0.87	619
	24	−***0.002***			***0.072***									***0.019***	0.85	512
	25										***0.012***	***0.016***	***0.006***		0.87	593
	26						***0.011***	***0.014***	−0.002						0.86	525
	27	−***0.002***		***0.134***		0.000									0.46	73
	28	−***0.004***			***0.179***	0.000									0.50	84
4	29	0.000					***0.012***	***0.014***	−0.002						0.86	394
	30	0.000									***0.013***	***0.016***	***0.006***		0.87	443
	31	−***0.002***	0.007	***0.065***										***0.019***	0.86	407
	32	−***0.003***	−***0.060***		***0.111***					***0.016***					0.88	482
	33	−***0.002***	−***0.093***	***0.128***						***0.017***					**0.89**	525
	34	−***0.002***	***0.049***		0.024									***0.020***	0.85	391
	35	−0.002	−***0.161***	***0.268***		0.000									0.49	60
	36	−***0.003***	−***0.216***		***0.377***	0.000									0.55	78
5	37	−***0.001***		***0.024***							***0.013***	***0.016***	***0.005***		0.87	362

Variables included in each model are indicated by their regression coefficient displayed in a white box (bold and italicized text indicates that it is a significant predictor with *p* value < 0.05)

**Table 3:** Multiple regression models for MPS with each row containing a unique set of predictor variables to form a model with the model adjusted coefficient of determination (Adj *R* ^2^) and *F* value in the right hand columns (bold text in adjusted *R* ^2^ and *F* columns indicate maximum values).

No. of variables	Model no.	Peak *g*	*V*_i_	Δ*V*_R_	*V*_R_	*α*_R_	Δ*ω*x	Δ*ω*y	Δ*ω*z	Δ*ω*_R_	*ω*x	*ω*y	*ω*z	*ω*_R_	Adj *R* ^2^	*F*
1	1	***0.003***													0.43	192
	2		***0.066***												0.45	206
	3			***0.062***											0.48	234
	4				***0.070***										0.51	260
	5									***0.011***					0.89	**2023**
	6													***0.014***	0.83	1239
	7					***0.000***									0.17	49
2	8	0.000		***0.060***											0.48	116
	9	−0.001			***0.088***										0.51	132
	10	0.000								***0.011***					0.89	1008
	11	***0.022***												***0.012***	0.85	735
	12			0.002						***0.011***					0.89	1009
	13			***0.020***										***0.012***	0.86	788
	14				***0.022***									***0.012***	0.86	772
	15				0.001					***0.011***					0.89	1008
	16	***0.003***				0.000									0.44	95
	17			***0.064***		0.000									0.49	116
	18				***0.074***	0.000									0.52	129
	19					0.000				***0.011***					0.89	1025
	20					***0.000***								***0.014***	0.84	640
3	21	−***0.001***		***0.014***						***0.011***					0.89	686
	22	0.000		***0.027***										***0.012***	0.86	526
	23	−0.001			0.013					***0.011***					0.89	679
	24	0.000			***0.026***									***0.012***	0.86	514
	25										***0.006***	***0.010***	***0.009***		0.89	720
	26						***0.006***	***0.008***	***0.003***						0.87	556
	27	0.989		***0.000***		0.838									0.49	77
	28	−0.001			***0.092***	0.000									0.52	87
4	29	0.000					***0.005***	***0.008***	0.002						0.87	417
	30	0.000									**0.006**	**0.010**	**0.008**		0.89	541
	31	0.000	0.022	0.009										***0.012***	0.86	397
	32	0.000	−0.022		***0.034***					***0.011***					0.89	514
	33	−0.001	−***0.036***	***0.044***						***0.011***					0.89	530
	34	0.000	***0.043***		−0.016									***0.013***	0.86	398
	35	0.000	−***0.083***	***0.134***		0.000									0.50	61
	36	−0.001	−***0.133***		***0.214***	0.000									0.56	78
5	37	***0.001***		−***0.019***							***0.006***	***0.010***	***0.009***		**0.90**	447

Variables included in each model are indicated by their regression coefficient displayed in a white box (bold and italicized text indicates that it is a significant predictor with *p* value < 0.05)

**Table 4:** Summary of the variables included in multiple regression models predicting CSDM-15 (top) and MPS (bottom) resulting in the greatest *F*-statistic.

Impact	No. of variables	Variables included in regression model	Adj *R* ^2^	*F*
*CSDM*-*15*				
All	1	***Δω***_***R***_	0.86	1629
Front		***Δω***_***R***_	0.83	522
Back		***Δω***_***R***_	0.96	2386
Side		***Δω***_***R***_	0.72	185
*MPS*				
All	1	***Δω***_***R***_	0.89	2023
Front		***Δω***_***R***_	0.83	479
Back		***Δω***_***R***_	0.93	1072
Side		***Δω***_***R***_	0.78	253

Each row represents one of the four impact group considerations: all impacts considered together (All) and individual impact locations (Front, Back, Side). Variables included in each model are indicated (bold and italicized text indicates that it is a significant predictor with *p* value < 0.05)

**Table 5:** Summary of the variables included in multiple regression models predicting CSDM-15 (top) and MPS (bottom) resulting in the greatest adjusted *R* ^2^.

Impacts	No. of Variables	Variables included in regression model												Adj *R* ^2^	*F*
*CSDM*-*15*															
All	4	***Peak g***	***V***_***i***_	***ΔV***_***R***_						***Δω***_***R***_				0.89	525
Front	4	Peak *g*									***ωx***	***ωy***	***ωz***	0.92	289
Back	2				***V***_***R***_					***Δω***_***R***_				0.98	2314
Side	2					*α*_R_				***Δω***_***R***_				0.82	157
*MPS*															
All	5	***Peak g***		***ΔV***_***R***_							***ωx***	***ωy***	***ωz***	0.90	447
Front	5	***Peak g***		***ΔV***_***R***_							***ωx***	***ωy***	***ωz***	0.93	283
Back	3						***Δωx***	***Δωy***	***Δωz***					0.94	473
Side	4	***Peak g***	*V*_i_	Δ*V*_R_		***α***_***R***_								0.82	73

Each row represents one of the four impact group considerations: all impacts considered together (All) and individual impact locations (Front, Back, Side). Variables included in each model are indicated (bold and italicized text indicates that it is a significant predictor with *p* value < 0.05)

**Table 6:** Summary of the variables included in the best two-variable regression models for predicting CSDM-15 (top) and MPS (bottom).

Impacts	No. of variables	Variables included in regression model			Adj *R* ^2^	*F*
*CSDM*-*15*						
All	_2_		***α***_***R***_	***Δω***_***R***_	0.87	851
Front			*α*_R_	***Δω***_***R***_	0.85	267
Back		***V***_***R***_		***Δω***_***R***_	0.98	2314
Side			*α*_R_	***Δω***_***R***_	0.82	157
*MPS*						
All	2		*α*_R_	***Δω***_***R***_	0.89	1025
Front			***α***_***R***_	***Δω***_***R***_	0.89	356
Back			***α***_***R***_	***Δω***_***R***_	0.94	612
Side			***α***_***R***_	***Δω***_***R***_	0.81	142

Each row represents one of the four impact group considerations: all impacts considered together (All) and individual impact locations (Front, Back, Side). Variables included in each model are indicated (bold and italicized text indicates that it is a significant predictor with *p* value < 0.05)

The location of MPS is reported in Table 7 by highlighting the element that experienced the greatest tensile strain during impact. MPS was most commonly reached in the cerebrum in regions adjacent to the cerebellum on the left and the right. Overall, more than 80% of maximum strains occurred in this region (88% of Front, 93% of Back, 65% for Side impacts).

**Table 7:** Location of SIMon-computed MPS for varying impact locations.

The single element containing MPS for each of the simulated impacts is considered here and all identified elements are highlighted

The single best kinematic predictor for both CSDM-15 and MPS considering all impact locations together was Δ*ω*_R_ as shown by *R* ^2^ of 0.86 and *F*-statistic 1629 (Table 2, row 5) for CSDM-15 and *R* ^2^ of 0.89 and *F*-statistic 2023 (Table 3, row 5) for MPS. Each kinematic variable was statistically significant in predicting CSDM-15 and MPS when acting as an individual predictor (Tables 2, 3, rows 1–7), though Δ*ω*_R_ identifies as the most efficient model for predicting both strain measures and a better option than peak *g* and *α*_R_. The highest adjusted *R* ^2^ for CSDM-15 was obtained using a 4-variable regression model including peak *g*, *V*_i_, Δ*V*_R_ and Δ*ω*_R_ (Table 2, row 33). A 5-variable model resulted in the highest adjusted *R* ^2^ value for MPS including peak *g*, Δ*V*_R_, *ω*_x_, *ω*_y_, and *ω*_z_ (Table 3, row 37). Considering all regressions models (Tables 2, 3, rows 1–37), as a new term was added to a previous model, adjusted *R* ^2^ increased or stayed the same.

For CSDM-15, as the number of predictor variables increased from one to four, the maximum *F*-statistic decreased from 1629 (row 5), to 851 (row 19), to 638 (row 21), to 525 (row 33). MPS noted similar trends. The primary reason for the decreasing trend in *F*-statistic was that adjusted *R* ^2^ only increased from 0.86 (row 5) to 0.89 (row 33) despite increasing the number of predictor variables from one to four. That is to say, the modest improvement in explained variance between models in row 5 and row 33 (0.86–0.89) required addition of three more predictor variables resulting in a less efficient regression model (as shown by low *F*-statistic).

To establish whether the findings associated with CSDM-15 and MPS are specific to helmet impact location, the preceding statistics were repeated considering data separately for front, back and side impact locations. All individual impact locations determined the best single kinematic predictor to be Δ*ω*_R_ for both CSDM-15 and MPS, resulting in the greatest *F*-statistic in all cases (Table 4). Furthermore, whether considering all impacts together or individual impact locations, Δ*ω*_R_ consistently resulted in stronger correlations than *α*_R_. In the case of back impacts, models predicting CSDM-15 achieved a maximum adjusted *R* ^2^ of 0.98 with *F*-statistic 2314 with a two-variable model including *V*_R_ and Δ*ω*_R_ (Table 5). For all other impact locations for both CSDM-15 and MPS, the greatest adjusted *R* ^2^ resulted from a model containing no fewer than three variables (Table 5).

## Discussion

This study used multiple regression techniques to determine the least number of kinematic terms necessary to predict brain strain calculated using SIMon for one configuration of test equipment.

This study proves that it is possible to create a model capable of predicting brain strain measures based on multiple linear and angular kinematics, though a single angular kinematic can predict both CSDM-15 and MPS. The single best kinematic predictor for CSDM-15 and MPS is Δ*ω*_R_, consistent when considering all impact locations together as well as when considering each impact location separately. In all cases, the model that achieved the highest *F*-statistic for predicting both brain strain measures included only Δ*ω*_R_. The indication that brain strain can be predicted using a single kinematic Δ*ω*_R_ agrees with work by Takhounts *et al*. who established angular velocity correlated better with CSDM and MPS than any other kinematic measures or functionals when considering injury in automotive impacts.^31^ Through the development of BrIC, Takhounts *et al*. confirmed that angular velocity is a better predictor for CSDM and MPS than angular acceleration and linear acceleration,^30^ which is in agreement with the findings in the present study as indicated by Δ*ω*_R_ showing a greater *R* ^2^ and *F*-statistic than peak *g* and *α*_R_.

**Figure 4:**
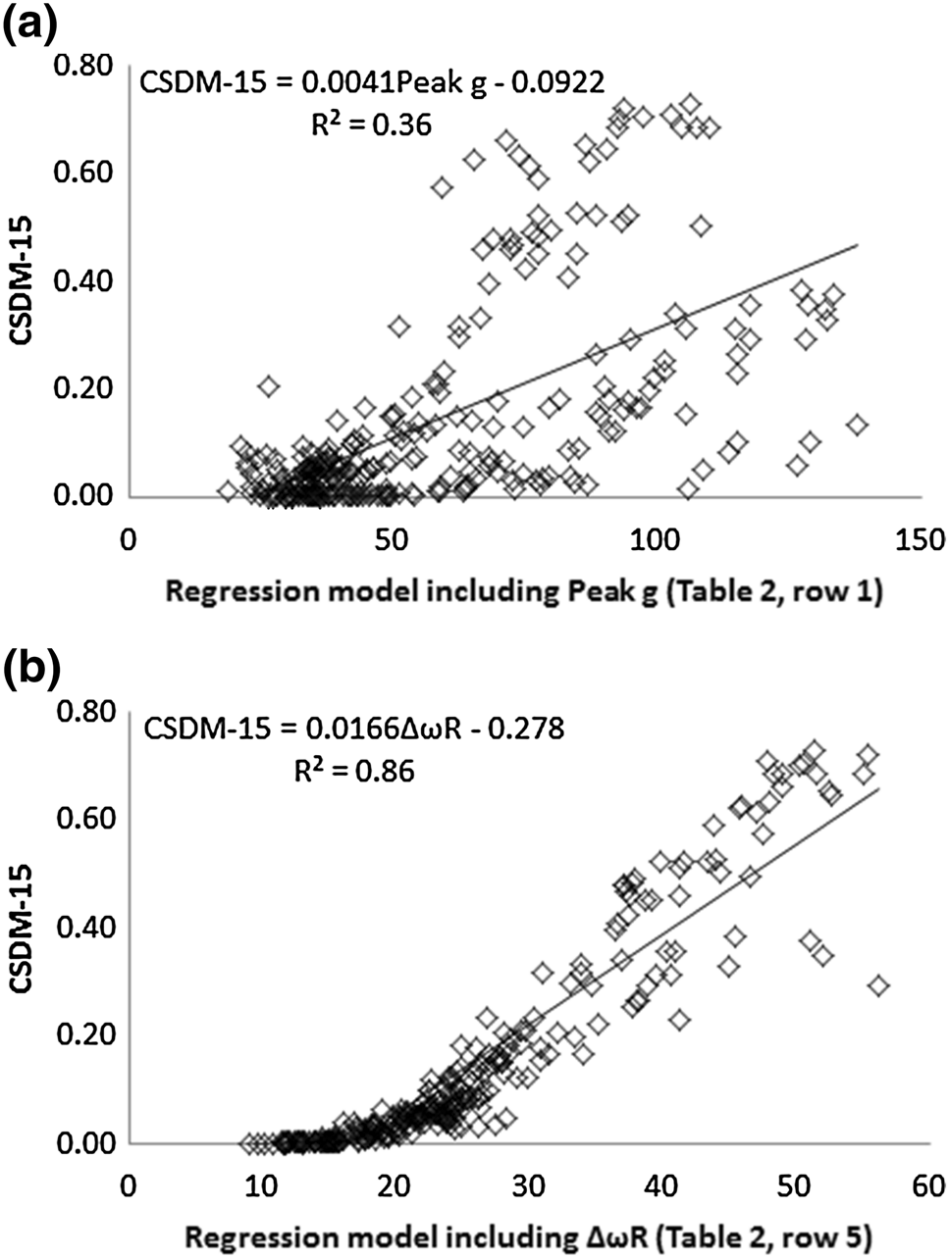
Regression models plotted against CSDM-15 for (a) a model containing peak *g* only and (b) a model including Δ*ω*_R_.

**Figure 5:**
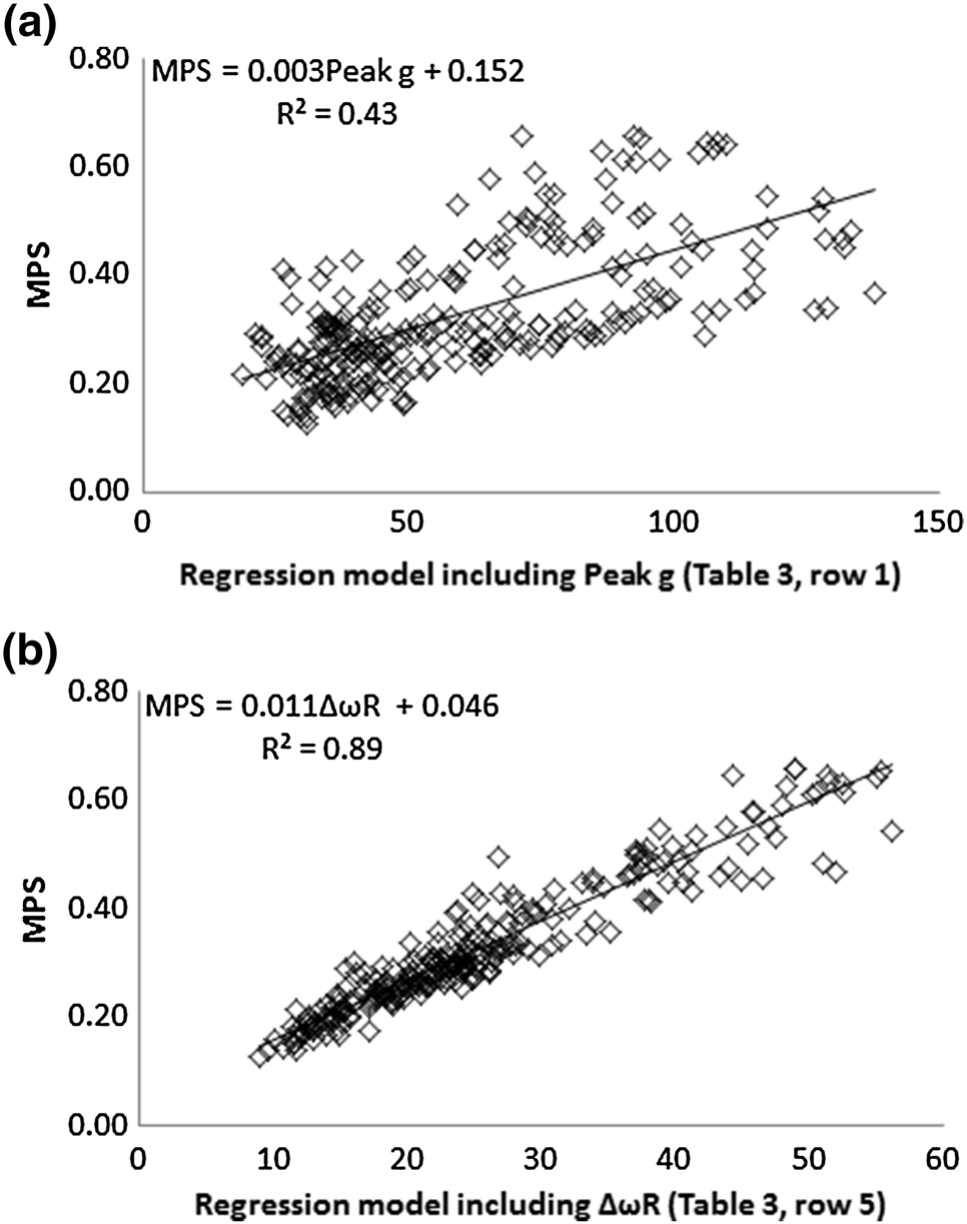
Regression models plotted against MPS for (a) a model containing peak *g* only and (b) a model including Δ*ω*_R_.

Considering single variable regression models, Δ*ω*_R_ better predicts CSDM-15 and MPS than the current helmet certification metric, peak *g*, as shown by the plots in Figs. 4 and 5. A regression model including Δ*ω*_R_ compared to a model containing peak *g* achieved greater adjusted *R* ^2^ and *F*-statistic. The significance of this finding is that as standard organizations discuss adopting new test methods to include angular motion, this work determines that the best method for predicting brain strain involves monitoring Δ*ω*_R_ rather than peak *g* alone.

Considering CSDM-15, provided Δ*ω*_R_ was included in the regression model, adjusted *R* ^2^ improved by a maximum 3.5%, when increasing the number of terms from one variable to four variables (Table 2, row 5 to row 33). For the same change in predictor variables (Table 2, row 5 to row 33), the *F*-statistic decreased by nearly 70%. A single-variable model is simple and capable of predicting brain strain measures; therefore, a more complex, multi-variable model may not be necessary to estimate diffuse injury of a certification-style drop test of a helmet.

In the case of both CSDM-15 and MPS, the model that maximizes adjusted *R* ^2^ does not align with the model that maximizes the *F*-statistic. The model with maximum adjusted *R* ^2^ for predicting CSDM-15 includes peak *g*, *V*_i_, Δ*V*_R_, and Δ*ω*_R_. The model with the highest adjusted *R* ^2^ for MPS includes peak *g*, Δ*V*_R_ and *ω*_x_, *ω*_y_, and *ω*_z_. Fewer variables create a more efficient model and maximize the *F*-statistic. Choosing a model with the highest adjusted *R* ^2^ could require measuring up to five different kinematic terms, though brain strain measures can be predicted with as little as one angular variable.

Considering impact data for impact locations separately (front, back, side), there is no agreement on a set of multiple kinematics that proves to be the best predictor for CSDM-15 and MPS. The best two-variable model for predicting CSDM-15 for front and side impacts includes *α*_R_ and Δ*ω*_R_, while back impacts favour a model that includes *V*_R_ and Δ*ω*_R_ (Table 5). For models containing three and four variables, each impact location shows a different set of kinematics forming the best model for predicting CSDM-15. Similar trends were found for MPS.

Considering all impact locations together, a two-variable model based on *α*_R_ and Δ*ω*_R_ achieves the greatest *R* ^2^ and *F*-statistic for predicting CSDM-15 (Table 2, row 19), and MPS (Table 3, row 19). Angular acceleration, *α*_R_, in combination with angular velocity creates an efficient and effective model for predicting both CSDM-15 and MPS, however, *α*_R_ in a model with any linear kinematics greatly reduces the efficiency as indicated by lower *F*-statistic. Including three or more kinematics in an attempt to improve *R* ^2^ gave no single set of kinematics that was the best for predicting strain measures. A regression model centered on Δ*ω*_R_ proves to be the single most efficient model to predict CSDM-15 and MPS.

**Figure 6:**
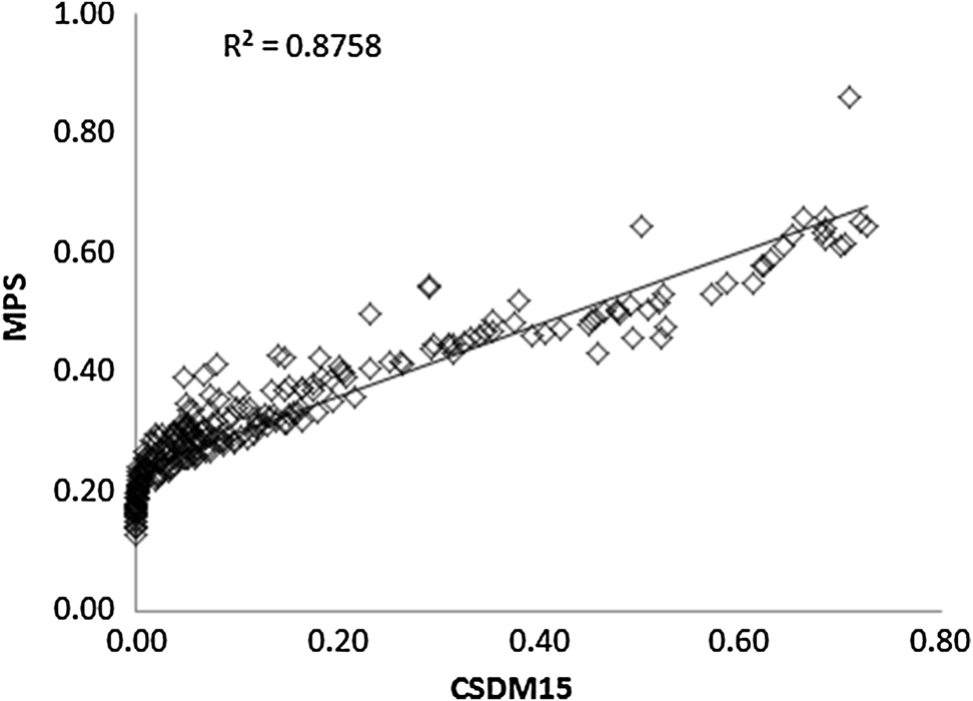
Maximum principal strain plotted against cumulative strain damage measure (specifically CSDM-15).

As CSDM-15 and MPS represent different methods for reporting brain strain, discussion surrounds which is more appropriate for representing brain injury risk during impact. CSDM-15 reports the volume of brain tissue that exceeds a maximum strain of 15%, while MPS reports the peak tensile strain experienced at any time during impact. As the lowest reported MPS was 0.13, CSDM-15 captures most of the MPS volumes present in this study. Plotting CSDM-15 against MPS, confirms the correlation between the two strain measures, and shows that higher MPS values are accompanied with appropriately increasing volumes reaching higher strain levels (Fig. 6). This suggests that kinematics capable of predicting either measure will be comparably accurate for representing potential injury risk during certification style helmeted impacts.

Should a new test protocol that includes head rotation be adopted, it will be necessary to distinguish variations in helmet performance. Ice hockey helmets capable of mitigating Δ*ω*_R_, for example, would then be favourable during helmet assessment protocols, based on the findings of this study.

**Figure 7:**
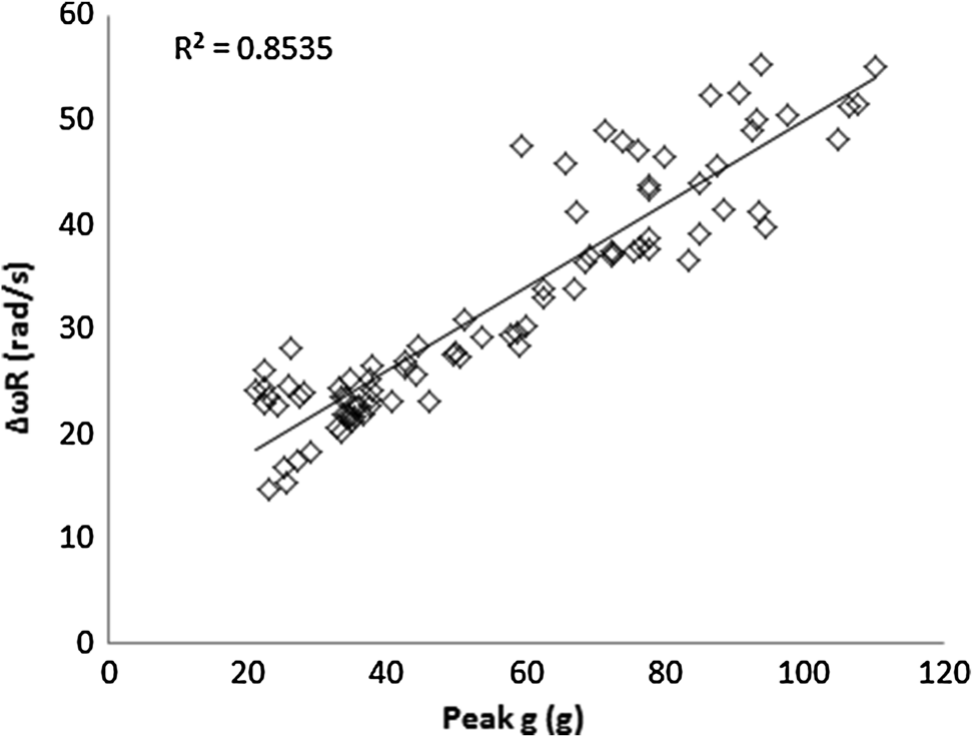
Resultant change in angular velocity, Δ*ω*_R_ plotted against peak *g* for impacts to the back of the helmet.

It is known that inputting only linear acceleration (absent of head rotation) into SIMon will result in near zero tissue strain, however, this work found that linear kinematics proved significant predictors of both CSDM-15 and MPS. The primary explanation for this is that, due to the presence of a neck model, linear kinematics correlate to angular kinematics and subsequently brain strain. The correlation between peak *g* and Δ*ω*_R_ is especially evident in back impacts as seen in Fig. 7. The primary implication of this finding is that the mechanics of the neck dictate headform rotation and consequently influence which kinematics are the best predictors of brain strain measures.

Due to the influence of the neck, for discussion on future assessment functions, the neck must be an ongoing consideration. It is agreed upon that the HybridIII neck response differs by rotation axes and until now it was unclear whether that variation would affect which kinematics relate to strain measures if used for helmet certification. While the neck plays a central role in influencing which kinematics best predict strain, this work proves that for testing protocols with the HybridIII head and neck, a single kinematic measure, Δ*ω*_R_, can predict strain measures for all impact locations. In parallel work, we will be documenting correlations for impact experiments without a neck to contribute to the ongoing debate regarding neck considerations.

One limitation of this work is that we used the SIMon model exclusively. It is known that the outputs of the SIMon model, on the basis of CSDM and MPS, correlate with the GHBMC,^30^ and therefore it may be possible to extrapolate our findings to other models. Ji *et al*. compared impact response variables of SIMon, the Dartmouth Scaled and Normalized Model (DSNM) and the Wayne State University Head Injury Model (WSUHIM), and found that while output variable magnitudes differed, all models showed correlation with peak linear and angular acceleration,^16^ though angular velocity was not considered in the study. Future work using additional models should be performed to confirm correlation efforts. The choice to use SIMon in this study is based on its validation process. Injury metrics were developed based on anatomic injury and accident reconstructions using the HybridIII head and neck, making its use, in combination with our test bed, the most appropriate choice. However, we acknowledge the possibility that the findings in this study could be altered by virtue of using another brain model.

This study is limited to relatively short impact durations associated with helmeted impacts. As we aim to focus on kinematics that predict strain measures specifically for helmeted impacts, this study did not consider impact durations exceeding 80 ms.

Further, we acknowledge that the use of the HybridIII neck influences the results of this study and the predictor variable coefficients in each regression model are dictated by our specific experimental setup. Future work will include helmeted impacts with the HybridIII head and no neck constraint to determine whether the findings in the present study change.

This study is the first to document impact location-specific results to determine the best kinematic predictors for brain strain measures. Hundreds of helmeted impacts were completed at varying locations and an extensive statistical analysis was performed showing conclusively that while individual kinematic parameters are statistically significant when considered alone, angular velocity is the single best kinematic predictor for brain strain and that a combination of linear and angular kinematics can predict brain strain measures. Considering independently the various impact locations, *α*_R_ and Δ*ω*_R_ were identified as the best two-variable model for strain prediction in nearly all cases, excluding only back impacts for CSDM-15 prediction. This study also shows that the mechanics of the neck will dictate the strains predicted, and therefore which kinematics predict brain strain. The results of this study could be used to inform which kinematics could be included for helmet assessment using drops with the HybridIII head and neck.
